# Sports and leisure coverage in Brazil: an analysis of the last 20 years

**DOI:** 10.3389/fspor.2024.1347212

**Published:** 2024-07-10

**Authors:** Ricardo Borges Viana, Rizia Rocha-Silva, Marcos José Morais, Victor Silveira Coswig, Mário Antônio de Moura Simim, Rodrigo Luiz Vancini, Marília Santos Andrade, Claudio Andre Barbosa de Lira

**Affiliations:** ^1^ Institute of Physical Education and Sports, Federal University of Ceará, Fortaleza, Brazil; ^2^ Faculty of Physical Education and Dance, Federal University of Goiás, Goiânia, Brazil; ^3^ Physical Education and Sports Center, Federal University of Espírito Santo, Vitória, Brazil; ^4^ Department of Physiology, Federal University of São Paulo, São Paulo, Brazil

**Keywords:** investments, sports, leisure, politics, public policy

## 1 Introduction

The recent Brazilian presidential elections held in 2022, won by Luíz Inácio Lula da Silva (Lula), raised various issues on social media concerning public policy, such as sports and leisure coverage (i.e., financial support). In addition, the Brazilian presidential elections were marked by intense polarization. As Brazil has several political ideologies (left-, center-, and right-wing), we investigated whether public financial support for sports and leisure has changed under different presidents between 2003 and 2022, which were as follows: Lula (2003–2010, left wing), Dilma Vana Rousseff (2011–2016, left wing), Michel Miguel Elias Temer Lulia (2016–2018, center wing), and Jair Messias Bolsonaro (2019–2022, right wing). The differentiation of political parties between the left wing and right wing is often based on their stance on the unidimensional role of state intervention in the economy and defense of social equality as a natural or constructed phenomenon (1). The center wing refers to a group of political parties that do not have a specific ideological orientation and whose objective is to ensure proximity to the executive power to guarantee their advantage and allow them to distribute privileges through clientele networks (2). This group is pejoratively called “*centrão*” (2).

These different political ideologies are reflected in the different policies implemented by different governments. Brazilian left-wing governments are characterized as welfare-friendly, whereas center- and right-wing governments are more neo-liberal and advocate less state participation. Regardless of political positioning, previous studies have aimed to evaluate the effects of public policies and different population outcomes. For example, numerous countries have implemented cash transfer public policies to combat poverty (3). Neves et al. (3) conducted a narrative review to analyze the contributions of the Brazilian Cash Transfer Program (named *Bolsa Família*), established in 2003, to reduce social inequalities and ensure the right to health, food, education, and social assistance. The results revealed a relationship between the Brazilian Cash Transfer Program, reduction in child mortality, increased access to primary healthcare services, increased access to food, higher school attendance, and reduced dropout rates. The study concluded that this program is a potent intersectoral policy for reducing inequities, which reinforces the need to strengthen and combine complementary policies to expand its effects. Thus, in 2022, during the COVID-19 pandemic, President Jair Bolsonaro's government created a cash transfer public policy, called “*Auxílio Emergencial*,” to mitigate the economic impacts caused by the COVID-19 pandemic in Brazil among the most vulnerable people, such as those who were unemployed or informally employed (4).

With regard to sports and leisure coverage, policies promoting sports and leisure play a crucial role in the development of a nation and the wellbeing of its citizens (5–13). More than providing just entertainment, these policies can significantly impact several areas, contributing to the strengthening of society and improving citizens’ quality of life (5–7). Moreover, many studies have highlighted the importance of these policies with regard to health and wellbeing (5), social inclusion (6, 7), economic development (8), education and values formation (9), national identity (10–12), and violence prevention (13). Therefore, effective implementation of policies aimed at sports and leisure enriches citizens’ quality of life and contributes to the development of a healthier, more cohesive, and prosperous society. Investment in these sectors reflects not only sporting achievements but also lasting benefits for the country's overall development.

Considering the political polarization that Brazil is experiencing and the importance of sports and leisure policies, a study investigating and comparing public financing in this area between governments with different ideological positions is necessary. Therefore, this study aimed to investigate sports and leisure coverage in Brazil over the last 20 years. Taking into account the distinct ideological stances among left, center, and right political parties, we hypothesized that left-wing administrations (Lula and Dilma) would exhibit higher levels of public investment in sports and leisure when contrasted with center (Temer) and right-wing governments (Bolsonaro).

## 2 Methods

### 2.1 Study design and procedures

This was a retrospective study that examined data regarding Brazilian public financial support for sports and leisure and Brazilian gross domestic product (GDP) between 2003 and 2022 available in online public databases called Advanced Management Budget Information System (*SIGA Brasil*) (14) and the Brazilian Institute of Geography and Statistics (15), respectively. We chose this timeline because before 2003 the available public information did not allow discrimination on Brazilian public financial support for sports and leisure.

The Brazilian Senate developed the *SIGA Brasil* web-based platform (14), aiming to investigate, supervise, and promote transparency of federal spending (16). We searched the platform using functions, subfunctions, and actions related to sports and leisure (based on the functional-programmatic classification). The platform search was performed through “open access,” using the tool “Web Intelligence,” leading to access to the “universe” where execution expenses are located. We saved data gathered about spending and the number of entries (i.e., completed individual actions) on sports and leisure coverage in the form of Microsoft Excel spreadsheets for further analysis. This search was conducted on 1 January 2023. Absolute annual sports and leisure coverage of each presidential term between 2003 and 2022 was corrected by the official Brazilian inflation index named the Brazilian Extended National Consumer Price Index (IPCA, *Índice Nacional de Preços ao Consumidor Amplo*) (17). Later, the data in Brazilian real (BRL, the Brazilian legal currency) were converted to US dollars (US$) based on the exchange rate on 18 March 2023 (1 US$ ≅ 5.2679 BRL) through the Brazilian Central Bank website (18). Subsequently, we determined the percentage of the GDP (%GDP) that was offered for sports and leisure coverage in each year between 2003 and 2022.

Furthermore, we also calculated the annual sports and leisure coverage per capita of each presidential term between 2003 and 2022, and annual sports and leisure coverage data were converted from BRL to US$ based on its respective annual average exchange rate through the Brazilian Central Bank website (19). The data in dollars were divided by the respective population size available on the Brazilian Institute of Geography and Statistics website (20). This study did not require approval from the Institutional Ethics Committee because it used publicly available data and did not involve human participants.

### 2.2 Statistical analyses

As all data were drawn directly from a true population rather than a scientific sample, we performed a descriptive analysis to present the Brazilian sports and leisure public financial support based on the “function” and “subfunction” codes for each presidential term between 2003 and 2022. The Shapiro–Wilk test was used to assess data distribution. As annual sports and leisure coverage data did not present a normal distribution, median and interquartile range (IQR) were used to present the annual sports and leisure coverage among the Lula first, Lula second, Dilma first, Dilma second, Temer, and Bolsonaro presidential terms (21). All statistical analyses were performed using Jeffreys's Amazing Statistics Program (version 0.18.3.0, University of Amsterdam, Netherlands).

## 3 Results

A descriptive analysis of public financial support for sports and leisure showed 734 entries between 2003 and 2022 (Supplementary Material 1). Lula first spent approximately $343 million in 4 years; Lula second spent approximately $1 billion in 4 years; Dilma first spent approximately $869 million in 4 years; Dilma second spent approximately $334 million in 2 years; Temer spent approximately $157 million in 2 years; and Bolsonaro spent approximately $174 million in 4 years (Supplementary Material 1). All of the presidential terms presented most of the entries about public financial support for sports and leisure under the function “sports and leisure” [Lula first (*n* = 326, 99.4%), Lula second (*n* = 152, 92.1%), Dilma first (*n* = 100, 92.6%), Dilma second (*n* = 40, 100%), Temer (*n* = 40, 97.6%), and Bolsonaro (*n* = 45, 86.5%)] (Supplementary Material 1). The main common subfunctions were community and performance sports for Lula first and Lula second presidential terms; community and performance sports, and general administration for Dilma first and Dilma second presidential terms; community and performance sports, and general administration for Temer presidential term; and community and performance sports, and worker protection and benefits for Bolsonaro presidential term.

**Figure 1:**
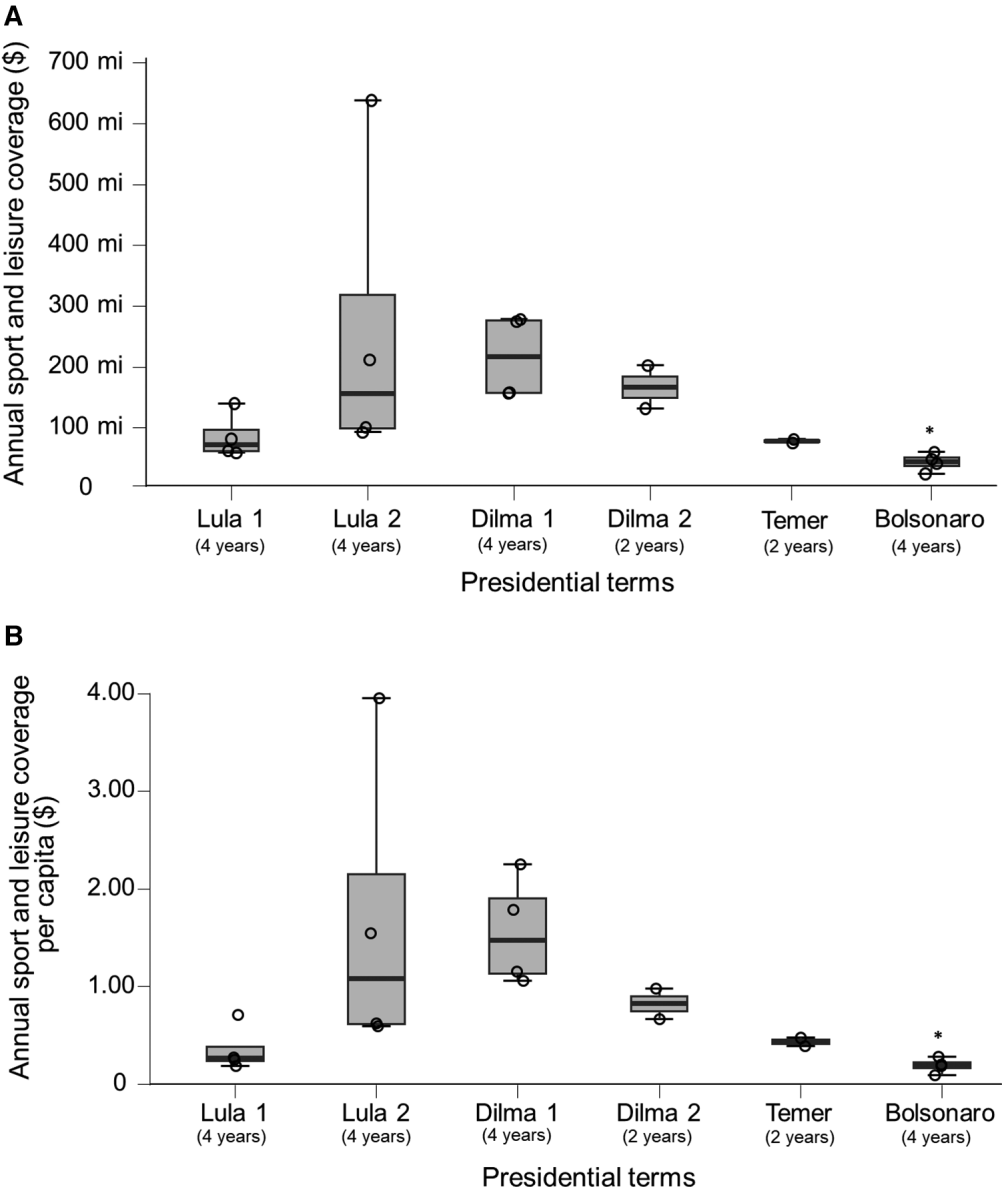
Absolute Brazilian annual sports and leisure coverage during presidential terms between 2003 and 2022 (**A**) and public financial support for sports and leisure per capita for each Brazilian presidential term (**B**).

**Figure 2:**
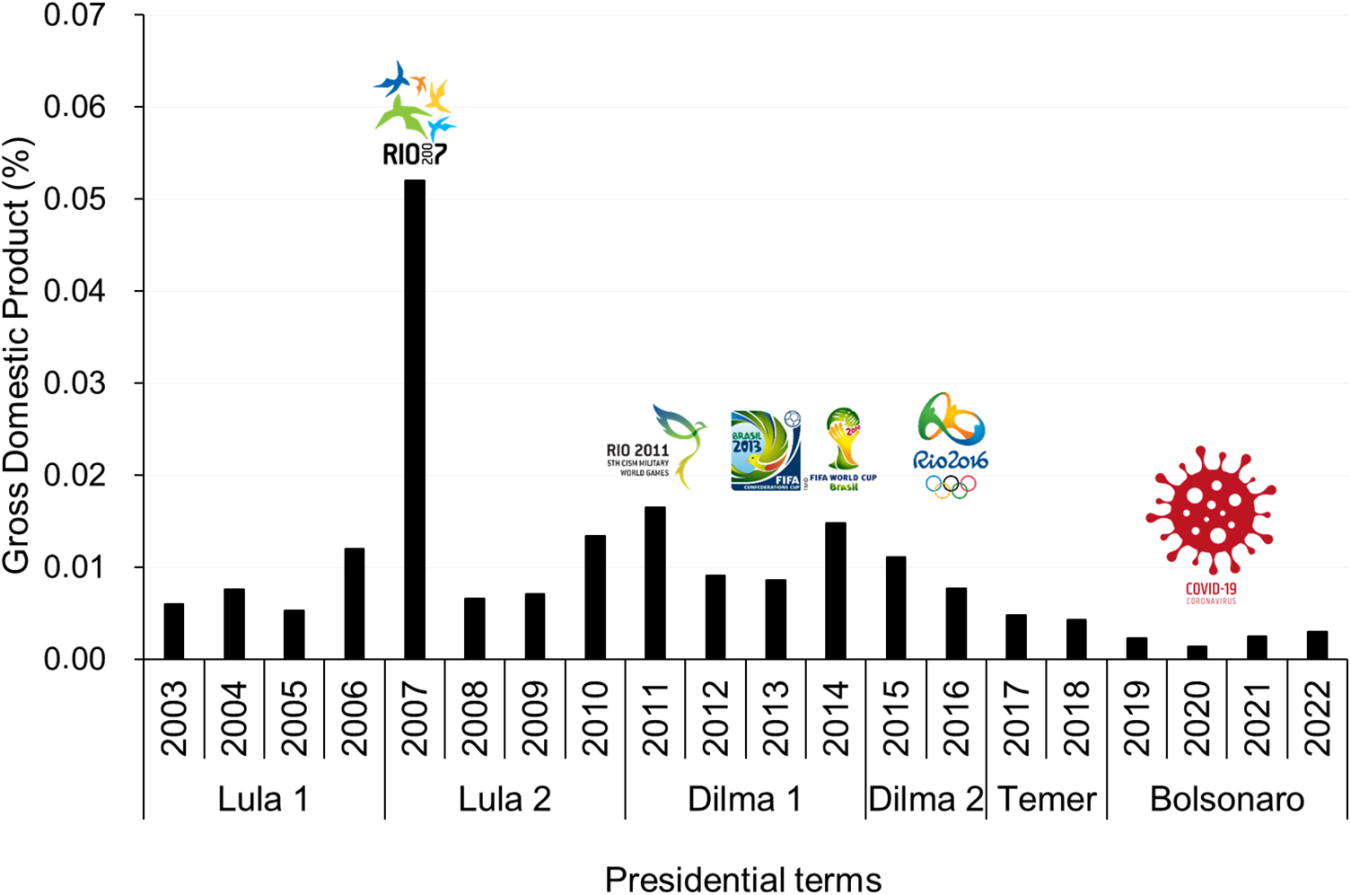
Percentage of Brazilian gross domestic product from 2003 to 2022 allocated to activities related to sport and leisure.

The Lula first, Lula second, Dilma first, Dilma second, Temer, and Bolsonaro presidential terms presented a median annual public financial support for sports and leisure of $72.1 million (IQR 34.5 million), $156.6 million (IQR 219.6 million), $216.7 million (IQR 11.8 million), $167.2 million (IQR 35.3 million), $78.3 million (IQR 3.0 million), and $44.6 million (IQR 1.4 million), respectively (Figure 1A). A descriptive analysis showed that the Lula first, Lula second, Dilma first, Dilma second, Temer, and Bolsonaro presidential terms presented a median annual public financial support for sports and leisure per capita of $0.27 (IQR 0.15), $1.09 (IQR 1.54), $1.47 (IQR 0.77), $0.83 (IQR 0.16), $0.44 (IQR 0.04), and $0.20 (IQR 0.06), respectively (Figure 1B). A descriptive analysis also showed that public investment in sports and leisure policies relativized by the Brazilian GDP presented higher values in international mega-sporting event years, particularly in 2007 (Figure 2).

## 4 Discussion

This study aimed to investigate sports and leisure coverage in Brazil over the last 20 years. As hypothesized, Brazilian presidential terms seem to affect annual public financial support for sports and leisure. Although these were objective and descriptive analysis results, some aspects may explain the differences in sports and leisure coverage in Brazil across presidential terms.

Mega-sport events may also be used as a strategy to secure additional funding from the national government for infrastructure development, funding that would not have been obtained in the absence of the event (22). In this context, several international mega-sporting events took place in Brazil between 2007 and 2016 (23, 24). For example, in 2007, during the first Lula presidential term, the Pan and Para Pan-American Games took place in Rio de Janeiro. Between 2011 and 2014, during the first Dilma presidential term, at least three important sports events took place in Brazil: the 5th Military World Games, hosted between 15 July and 24 July 2011 in Rio de Janeiro; the 9th FIFA Confederations Cup, hosted in six different Brazilian cities (Rio de Janeiro, Belo Horizonte, Fortaleza, Brasília, Salvador, and Recife) between 15 June and 30 June 2013; and the 20th FIFA World Cup for men's national football, hosted in 12 different Brazilian cities (Rio de Janeiro, São Paulo, Belo Horizonte, Brasília, Cuiabá, Curitiba, Fortaleza, Manaus, Natal, Porto Alegre, Recife, and Salvador) between 12 June and 13 July 2014 (Figure 2). Moreover, during the second Dilma presidential term, Rio de Janeiro hosted the Olympic and Paralympic Games 2016 (Figure 2). However, during the next two presidential elections (Temer and Bolsonaro), no mega-sporting events were hosted in Brazil, explaining the low sports coverage.

Although Brazil hosted many sports mega-events between 2007 and 2016, it is important to highlight that hosting such events in a country may not influence economic growth. Kobierecki and Pierzgalski (23) conducted a quasi-experimental study to tests the hypothesis that there is a positive causal effect of the Olympic Games and FIFA World Cup held between 2010 and 2016 on the economic development of Canada, South Africa, Great Britain, and Brazil. The authors found no significant effect of hosting sports mega-events on economic (i.e., GDP) growth of these countries. Therefore, we need to analyze cautiously the relationship between hosting sports mega-events and economic growth because the first one may not significantly influence the second one. However, we are aware of the fact that the costs and benefits of staging sports mega-events are not only about the tangible benefits, but also about intangible benefits, such as easing visa requirements, enhanced international awareness, urban regeneration, and increased sports achievements (23, 25).

In addition, the COVID-19 pandemic occurred during Bolsonaro's presidential term, which adversely affected all sports events worldwide due to strict healthcare measures (social isolation and interruption of activities considered non-essential) (26), consequently declining many possibilities to boost the Brazilian sports scenario. Furthermore, since 2016, Brazil has been facing significant economic difficulties that decreased public funding in education (27), science (28), technology (28), and health (29), which worsened because of the COVID-19 pandemic. Thus, President Jair Bolsonaro's government created a cash transfer program for people experiencing social vulnerability. Therefore, all of these factors may be related to the decrease in public investment in sports and leisure.

Notably, between 2003 and 2018, sports budgets were funded by the Brazilian Ministry of Sports (*Ministério do Esporte*), which held permanent ministry status. In January 2019, with the beginning of the Jair Bolsonaro government, the Brazilian Ministry of Sports was transformed into a Special Secretariat of Sports (*Secretaria Especial do Esporte*) within the newly established Ministry of Citizenship (*Ministério da Cidadania*). This restructuring caused internal disputes between the sport and other secretariats, impacting political, budgetary, and structural space.

In 2017, the mean public spending on sports and leisure by the 28 member countries of the European Union was €100 per inhabitant (30), where the highest and lowest recreation and sports government expenditure per inhabitant among the European Union member states were Luxembourg (€492) and Croatia (€13), respectively (30). In contrast, when BRL was converted to Euro based on the average exchange rate in 2017 (1 EUR ≅ 3.6098 BRL) (31), the Brazilian public spending on sports and leisure was only €0.42 (approximately $0.48) per inhabitant. Furthermore, the coverage was far below that of expenditure in developed countries, even in the years with the highest annual Brazilian public spending on sports and leisure per capita (2007, $3.95; 2011, $2.25; and 2014, $1.79) (30). In relative terms (percentage of the GDP), the lowest ratio of government recreation and sports expenditures to total expenditures among member countries of the European Union ranged from 0.2% in Croatia to 2.5% in Hungary in 2017, whereas it was 0.005% in Brazil (Figure 1B), indicating that Brazilian investment is below that practiced by developed countries. Such data are extremely worrying considering that public policies are an important tool for improving the population's quality of life.

In general, government funding plays a pivotal role in sustaining and ensuring the success of public policy initiatives. Consistent and reliable financial commitment from the government is imperative for the effective implementation, maintenance, and impact of policies that address pressing societal needs. Moreover, consistent government funding provides stability and fosters the long-term sustainability of public policies. It allows for establishing infrastructure, training programs, and institutional frameworks necessary for the effective delivery of services over time. In the present study, we found that after the first Dilma presidential term, there was a progressive decline in both absolute and relative (per capita) Brazilian annual sport and leisure coverage (Figure 1). Thus, it is time for the Brazilian government and people involved in the creation of sports politics to scrutinize sports promotion in Brazil, overcome old beliefs, and move forward.

This study has some limitations. First, we must consider that Brazil is a developing country that is chronically subjected to economic and/or political crises. Second, Brazil is a federal republic; thus, some investments may be made by states and funds coming from the states and municipalities. However, we did not evaluate these data. Third, we did not evaluate the health budget allocated to programs to encourage physical activity. Fourth, analyzing the budget allocated to mega-sporting events was not possible. Fifth, in terms of holding office, 14 years were associated with the left wing, 2 years with the center wing, and 4 years with the right wing. Thus, given the predominance of the left wing, our findings should be interpreted cautiously. The strength of this manuscript is that we used official data from the Brazilian federal government, which is reliable.

In conclusion, the presented brief research report showed that presidential terms affect annual public financial support for sports and leisure. However, the occurrence of many international mega-sporting events and the COVID-19 pandemic in some specific Brazilian presidential terms may have biased the analysis, and caution is needed when interpreting these results.
