# Supplementary material for: Sports and leisure coverage in Brazil: an analysis of the last 20 years
Source: Front Sports Act Living. 2024 Jul 10;6:1347212. doi: 10.3389/fspor.2024.1347212 (PMC11266043; doi:10.3389/fspor.2024.1347212)
Supplement: Supplementary file 2 [file Datasheet2.docx]

**Supplementary material 1.** Sport and leisure coverage in Brazil from 2003 to 2022.

| Presidential term | Year | Function (Code/Description) | Subfunction (Code/Description) | Number of entries | Coverage* |
| --- | --- | --- | --- | --- | --- |
| Lula 1 | 2003 | 27 - Sport and Leisure (n = 200) | 121 - Planning and budget | 1 | $ 152.646,23 |
|  |  |  | 128 - Human resources qualification | 3 | $ 167.157,40 |
|  |  |  | 421 - Custody and social reintegration | 2 | $ 5.821.611,65 |
|  |  |  | 572 - Technological development and engineering | 1 | $ 38.705,42 |
|  |  |  | 573 - Dissemination of scientific and technological knowledge | 1 | $ 165.180,82 |
|  |  |  | 811 - Performance sport | 7 | $ 3.806.519,82 |
|  |  |  | 812 - Community sport | 185 | $ 52.423.252,07 |
|  | 2004 | 27 - Sport and Leisure (n = 40) | 121 - Planning and budget | 1 | $ 422.691,25 |
|  |  |  | 122 - General administration | 2 | $ 12.736.674,63 |
|  |  |  | 128 - Human resources qualification | 3 | $ 240.480,47 |
|  |  |  | 131 - Social communication | 1 | $ 522.615,81 |
|  |  |  | 301 - Primary care | 1 | $ 82.653,42 |
|  |  |  | 306 - Food and nutrition | 1 | $ 174.721,55 |
|  |  |  | 331 - Worker protection and benefits | 1 | $ 88.238,20 |
|  |  |  | 365 - Child education | 1 | $ 18.959,90 |
|  |  |  | 811 - Performance sport | 13 | $ 23.345.233,84 |
|  |  |  | 812 - Community sport | 16 | $ 43.976.358,38 |
|  | 2005 | 12 - Education (n = 1) | 813 - Leisure | 1 | $ 2.561.524,23 |
|  |  | 27 - Sport and Leisure (n = 44) | 121 - Planning and budget | 1 | $ 281.255,36 |
|  |  |  | 122 - General administration | 2 | $ 11.994.717,61 |
|  |  |  | 126 - Information technology | 1 | $ 6.659,96 |
|  |  |  | 128 - Human resources qualification | 3 | $ 160.254,31 |
|  |  |  | 131 - Social communication | 1 | $ 557.291,77 |
|  |  |  | 301 - Primary care | 1 | $ 129.775,35 |
|  |  |  | 306 - Food and nutrition | 1 | $ 175.439,63 |
|  |  |  | 331 - Worker protection and benefits | 1 | $ 82.097,61 |
|  |  |  | 365 - Child education | 1 | $ 17.360,22 |
|  |  |  | 573 - Dissemination of scientific and technological knowledge | 1 | $ 39.483,33 |
|  |  |  | 811 - Performance sport | 14 | $ 5.635.675,27 |
|  |  |  | 812 - Community sport | 17 | $ 37.446.843,58 |
|  | 2006 | 12 - Education (n = 1) | 813 - Leisure | 1 | $ 9.481.088,59 |
|  |  | 27 - Sport and Leisure (n = 42) | 121 - Planning and budget | 1 | $ 1.159.825,39 |
|  |  |  | 122 - General administration | 4 | $ 17.624.403,06 |
|  |  |  | 128 - Human resources qualification | 3 | $ 736.256,72 |
|  |  |  | 131 - Social communication | 1 | $ 1.430.507,91 |
|  |  |  | 301 - Primary care | 1 | $ 127.316,00 |
|  |  |  | 306 - Food and nutrition | 1 | $ 165.558,54 |
|  |  |  | 331 - Worker protection and benefits | 1 | $ 100.204,38 |
|  |  |  | 365 - Child education | 1 | $ 14.965,01 |
|  |  |  | 573 - Dissemination of scientific and technological knowledge | 2 | $ 127.744,50 |
|  |  |  | 811 - Performance sport | 11 | $ 58.513.510,83 |
|  |  |  | 812 - Community sport | 16 | $ 50.648.353,39 |
| Lula 2 | 2007 | 12 - Education (n = 1) | 813 - Leisure | 1 | $ 438.587,43 |
|  |  | 27 - Sport and Leisure (n = 39) | 121 - Planning and budget | 1 | $ 239.269,37 |
|  |  |  | 122 - General administration | 3 | $ 23.705.007,68 |
|  |  |  | 128 - Human resources qualification | 3 | $ 1.144.218,55 |
|  |  |  | 131 - Social communication | 1 | $ 5.413.893,02 |
|  |  |  | 301 - Primary care | 1 | $ 113.507,32 |
|  |  |  | 306 - Food and nutrition | 1 | $ 126.574,78 |
|  |  |  | 331 - Worker protection and benefits | 1 | $ 69.384,14 |
|  |  |  | 365 - Child education | 1 | $ 8.769,86 |
|  |  |  | 573 - Dissemination of scientific and technological knowledge | 2 | $ 113.344,00 |
|  |  |  | 811 - Performance sport | 11 | $ 357.062.200,83 |
|  |  |  | 812 - Community sport | 13 | $ 248.664.688,07 |
|  |  |  | 813 - Leisure | 1 | $ 1.652.223,69 |
|  | 2008 | 12 - Education (n = 1) | 813 - Leisure | 1 | $ 1.124.316,04 |
|  |  | 27 - Sport and Leisure (n = 38) | 121 - Planning and budget | 1 | $ 477.105,10 |
|  |  |  | 122 - General administration | 3 | $ 20.328.854,34 |
|  |  |  | 128 - Human resources qualification | 3 | $ 1.093.490,64 |
|  |  |  | 131 - Social communication | 1 | $ 934.274,72 |
|  |  |  | 301 - Primary care | 1 | $ 107.244,36 |
|  |  |  | 306 - Food and nutrition | 1 | $ 181.313,09 |
|  |  |  | 331 - Worker protection and benefits | 1 | $ 78.221,64 |
|  |  |  | 365 - Child education | 1 | $ 14.954,10 |
|  |  |  | 811 - Performance sport | 9 | $ 19.122.459,23 |
|  |  |  | 812 - Community sport | 16 | $ 48.832.894,21 |
|  |  |  | 813 - Leisure | 1 | $ 613.071,56 |
|  | 2009 | 05 - National Defense (n = 5) | 811 - Performance sport | 5 | $ 34.980.990,78 |
|  |  | 12 - Education (n = 1) | 813 - Leisure | 1 | $ 889.182,53 |
|  |  | 27 - Sport and Leisure (n = 35) | 121 - Planning and budget | 1 | $ 1.634.754,82 |
|  |  |  | 122 - General administration | 3 | $ 21.022.538,78 |
|  |  |  | 126 - Information technology | 1 | $ 1.776.065,83 |
|  |  |  | 128 - Human resources qualification | 3 | $ 564.709,42 |
|  |  |  | 131 - Social communication | 1 | $ 1.746.184,40 |
|  |  |  | 301 - Primary care | 1 | $ 86.907,91 |
|  |  |  | 306 - Food and nutrition | 1 | $ 186.514,13 |
|  |  |  | 331 - Worker protection and benefits | 1 | $ 67.553,90 |
|  |  |  | 365 - Child education | 1 | $ 14.277,22 |
|  |  |  | 573 - Dissemination of scientific and technological knowledge | 1 | $ 3.394,40 |
|  |  |  | 811 - Performance sport | 5 | $ 5.761.375,37 |
|  |  |  | 812 - Community sport | 15 | $ 32.131.800,15 |
|  |  |  | 813 - Leisure | 1 | $ 280.009,84 |
|  | 2010 | 05 - National Defense (n = 5) | 811 - Performance sport | 5 | $ 107.242.572,46 |
|  |  | 27 - Sport and Leisure (n = 40) | 121 - Planning and budget | 1 | $ 4.956.643,98 |
|  |  |  | 122 - General administration | 3 | $ 29.077.655,57 |
|  |  |  | 128 - Human resources qualification | 3 | $ 1.165.135,49 |
|  |  |  | 131 - Social communication | 1 | $ 1.468.253,46 |
|  |  |  | 301 - Primary care | 1 | $ 119.804,87 |
|  |  |  | 306 - Food and nutrition | 1 | $ 435.115,36 |
|  |  |  | 331 - Worker protection and benefits | 1 | $ 89.563,36 |
|  |  |  | 365 - Child education | 1 | $ 13.201,93 |
|  |  |  | 573 - Dissemination of scientific and technological knowledge | 1 | $ 150.461,85 |
|  |  |  | 811 - Performance sport | 10 | $ 26.925.582,81 |
|  |  |  | 812 - Community sport | 15 | $ 36.073.223,63 |
|  |  |  | 813 - Leisure | 1 | $ 234.920,92 |
|  |  |  | 846 - Other special costs | 1 | $ 4.072.698,80 |
| Dilma 1 | 2011 | 05 - National Defense (n = 5) | 811 - Performance sport | 5 | $ 113.357.148,81 |
|  |  | 12 - Education (n = 1) | 812 - Community sport | 1 | $ 45.050.846,01 |
|  |  | 27 - Sport and Leisure (n = 35) | 121 - Planning and budget | 1 | $ 1.558.452,28 |
|  |  |  | 122 - General administration | 3 | $ 26.544.093,26 |
|  |  |  | 128 - Human resources qualification | 2 | $ 330.887,23 |
|  |  |  | 131 - Social communication | 1 | $ 8.554.158,83 |
|  |  |  | 301 - Primary care | 1 | $ 163.149,03 |
|  |  |  | 306 - Food and nutrition | 1 | $ 401.402,94 |
|  |  |  | 331 - Worker protection and benefits | 1 | $ 64.488,34 |
|  |  |  | 365 - Child education | 1 | $ 12.670,61 |
|  |  |  | 811 - Performance sport | 10 | $ 50.255.778,41 |
|  |  |  | 812 - Community sport | 13 | $ 32.256.856,93 |
|  |  |  | 813 - Leisure | 1 | $ 69.935,21 |
|  | 2012 | 13 - Culture (n = 1) | 813 - Leisure | 1 | $ 21.875.941,73 |
|  |  | 27 - Sport and Leisure (n = 25) | 121 - Planning and budget | 1 | $ 622.105,97 |
|  |  |  | 122 - General administration | 4 | $ 63.712.675,39 |
|  |  |  | 128 - Human resources qualification | 3 | $ 483.582,43 |
|  |  |  | 131 - Social communication | 1 | $ 9.524.254,18 |
|  |  |  | 301 - Primary care | 2 | $ 173.197,57 |
|  |  |  | 306 - Food and nutrition | 1 | $ 359.931,29 |
|  |  |  | 331 - Worker protection and benefits | 1 | $ 46.914,85 |
|  |  |  | 365 - Child education | 1 | $ 11.593,84 |
|  |  |  | 811 - Performance sport | 7 | $ 50.669.225,13 |
|  |  |  | 812 - Community sport | 4 | $ 10.601.246,65 |
|  | 2013 | 13 - Culture (n = 1) | 813 - Leisure | 1 | $ 20.669.158,60 |
|  |  | 27 - Sport and Leisure (n = 20) | 122 - General administration | 3 | $ 24.072.955,69 |
|  |  |  | 131 - Social communication | 1 | $ 8.194.054,34 |
|  |  |  | 301 - Primary care | 1 | $ 130.677,78 |
|  |  |  | 306 - Food and nutrition | 1 | $ 427.057,91 |
|  |  |  | 331 - Worker protection and benefits | 1 | $ 45.348,79 |
|  |  |  | 365 - Child education | 1 | $ 10.855,12 |
|  |  |  | 811 - Performance sport | 8 | $ 84.762.256,54 |
|  |  |  | 812 - Community sport | 4 | $ 18.833.045,96 |
|  | 2014 | 27 - Sport and Leisure (n = 20) | 122 - General administration | 3 | $ 27.853.120,21 |
|  |  |  | 131 - Social communication | 1 | $ 10.083.268,27 |
|  |  |  | 301 - Primary care | 1 | $ 35.725,44 |
|  |  |  | 331 - Worker protection and benefits | 4 | $ 457.979,12 |
|  |  |  | 811 - Performance sport | 7 | $ 209.343.975,14 |
|  |  |  | 812 - Community sport | 4 | $ 27.446.573,86 |
| Dilma 2 | 2015 | 27 - Sport and Leisure (n = 20) | 122 - General administration | 3 | $ 30.908.906,39 |
|  |  |  | 131 - Social communication | 1 | $ 5.079.119,31 |
|  |  |  | 301 - Primary care | 1 | $ 80.477,89 |
|  |  |  | 331 - Worker protection and benefits | 4 | $ 464.219,45 |
|  |  |  | 811 - Performance sport | 7 | $ 137.180.102,65 |
|  |  |  | 812 - Community sport | 4 | $ 28.807.188,73 |
|  | 2016 | 27 - Sport and Leisure (n = 20) | 122 - General administration | 4 | $ 28.486.870,01 |
|  |  |  | 131 - Social communication | 1 | $ 3.460.017,32 |
|  |  |  | 301 - Primary care | 1 | $ 209.368,93 |
|  |  |  | 331 - Worker protection and benefits | 3 | $ 510.823,47 |
|  |  |  | 811 - Performance sport | 7 | $ 78.727.669,98 |
|  |  |  | 812 - Community sport | 4 | $ 20.581.743,65 |
| Temer | 2017 | 27 - Sport and Leisure (n = 21) | 122 - General administration | 4 | $ 28.760.321,49 |
|  |  |  | 131 - Social communication | 1 | $ 1.729.932,45 |
|  |  |  | 301 - Primary care | 1 | $ 118.205,30 |
|  |  |  | 331 - Worker protection and benefits | 4 | $ 430.728,45 |
|  |  |  | 811 - Performance sport | 7 | $ 14.751.415,38 |
|  |  |  | 812 - Community sport | 3 | $ 35.056.394,74 |
|  |  |  | 846 - Other special costs | 1 | $ 434.897,40 |
|  | 2018 | 05 - National Defense (n = 1) | 811 - Performance sport | 1 | $ 1.615.324,53 |
|  |  | 27 - Sport and Leisure (n = 19) | 122 - General administration | 3 | $ 29.156.967,93 |
|  |  |  | 131 - Social communication | 1 | $ 1.416.151,33 |
|  |  |  | 301 - Primary care | 1 | $ 173.144,24 |
|  |  |  | 331 - Worker protection and benefits | 1 | $ 496.866,27 |
|  |  |  | 811 - Performance sport | 8 | $ 20.878.493,60 |
|  |  |  | 812 - Community sport | 4 | $ 21.099.908,95 |
|  |  |  | 846 - Other special costs | 1 | $ 417.913,18 |
| Bolsonaro | 2019 | 05 - National Defense (n = 1) | 811 - Performance sport | 1 | $ 2.232.813,08 |
|  |  | 27 - Sport and Leisure (n = 14) | 122 - General administration | 2 | $ 1.966.552,10 |
|  |  |  | 301 - Primary care | 1 | $ 2.406,08 |
|  |  |  | 331 - Worker protection and benefits | 1 | $ 42.933,65 |
|  |  |  | 811 - Performance sport | 7 | $ 29.387.148,29 |
|  |  |  | 812 - Community sport | 3 | $ 7.321.007,57 |
|  | 2020 | 05 - National Defense (n = 2) | 811 - Performance sport | 1 | $ 461.087,15 |
|  |  |  | 812 - Community sport | 1 | $ 432.718,44 |
|  |  | 27 - Sport and Leisure (n = 11) | 811 - Performance sport | 8 | $ 16.016.984,94 |
|  |  |  | 812 - Community sport | 3 | $ 7.496.142,67 |
|  | 2021 | 05 - National Defense (n = 2) | 811 - Performance sport | 1 | $ 594.203,92 |
|  |  |  | 812 - Community sport | 1 | $ 408.707,44 |
|  |  | 27 - Sport and Leisure (n = 10) | 811 - Performance sport | 7 | $ 26.433.236,65 |
|  |  |  | 812 - Community sport | 3 | $ 20.855.839,72 |
|  | 2022 | 05 - National Defense (n = 2) | 811 - Performance sport | 1 | $ 673.878,33 |
|  |  |  | 812 - Community sport | 1 | $ 566.009,15 |
|  |  | 27 - Sport and Leisure (n = 10) | 811 - Performance sport | 7 | $ 29.592.310,18 |
|  |  |  | 812 - Community sport | 3 | $ 29.261.584,74 |

*Data in Real (the Brazilian legal tender) were downloaded from the Advanced Management Budget Information System (*SIGA Brasil*), followed by a conversion to Dollar (1 Dollar = 5.2679 Reais) and corrected by the Brazilian Extended National Consumer Price Index (*Índice Nacional de Preços ao Consumidor Amplo* – IPCA).
